# Extracranial Internal Carotid Aneurysm Manifesting With Tinnitus: A Rare Presentation

**DOI:** 10.7759/cureus.21349

**Published:** 2022-01-17

**Authors:** Aadya Pillai, Poonam Arora, Reshma Asokan, Naveen Joseph, Silpa S

**Affiliations:** 1 Department of Emergency Medicine, All India Institute of Medical Sciences, Rishikesh, IND

**Keywords:** iatrogenic trauma, peritonsillar mass, internal carotid artery, extracranial aneurysm, pulsatile tinnitus

## Abstract

A 19-year-old male presented with a history of pulsatile tinnitus on the left side after he underwent drainage for a tonsillar swelling. He had noticed a painless neck swelling one month ago that was associated with difficulty in swallowing. His physician had made a diagnosis of peritonsillar abscess, and drainage had been performed. After the procedure, the swelling had progressed further, leading to drooling of saliva and disabling tinnitus. When he presented to the emergency, local examination revealed a pulsatile neck swelling with peritonsillar mass. CT angiography/digital subtraction angiography (DSA) of the neck and brain revealed a large aneurysm of the distal cervical segment of the left internal carotid artery (ICA). The patient successfully underwent an urgent open repair. This case highlights the importance of a thorough history and examination in identifying a rare cause of pulsatile tinnitus. A stepwise approach to determine the reason behind tinnitus revealed an underlying ICA aneurysm of the extracranial segment.

## Introduction

Pulsatile tinnitus can have a vascular or non-vascular etiology. The most common vascular causes include a high jugular bulb, glomus tumor, cerebral aneurysm of the internal carotid and vertebral-basilar arteries, and dural arteriovenous fistula [1]. The non-vascular etiologies include metabolic conditions causing increased cardiac output like pregnancy, anemia and thyrotoxicosis, myoclonus of palate, and middle ear musculature [2]. Pulsatile tinnitus has never been reported as a presenting feature of an aneurysm of the extracranial part of the internal carotid artery (ICA).

## Case presentation

A 19-year-old man with no past medical and surgical history presented to the emergency department with a history of a ringing sensation in the ear for the last seven days. The patient had a history of painless swelling in the neck as well as oral swelling on the left side with difficulty in speech for one month. He had consulted a local practitioner, where he had been diagnosed as a case of peritonsillar abscess, and drainage had been done. Since then, he had developed a ringing sensation in the left ear, which was pulsatile, followed by hoarseness of voice and difficulty in turning the neck towards the left side. There was no preceding history of fever, headache, vomiting, trauma, or drug intake, which could be responsible for pulsatile tinnitus. On physical examination, the patient had a pulsatile swelling on the left side of the neck, no bruit on auscultation, and no local rise in temperature. Oral examination showed an erythematous swelling in the left peritonsillar area. Central nervous system examination showed that uvula was deviated to the right side with absent gag reflex on the left side, weakness of the left sternocleidomastoid and trapezius muscle, and deviation of the tongue towards the left side (affection of left IX, X, XI, XII cranial nerves). The cardiovascular examination did not reveal any murmur or added sound on auscultation. Normal blood pressure was noted and was equal in both upper limbs. Ear examination revealed normal external ears and a shiny tympanic membrane on both sides with no redness or pulsation. Rinne’s, Weber’s tests, and an audiogram were suggestive of sensorineural hearing loss on the left side; however, this was unnoticed by the patient.

**Figure 1:**
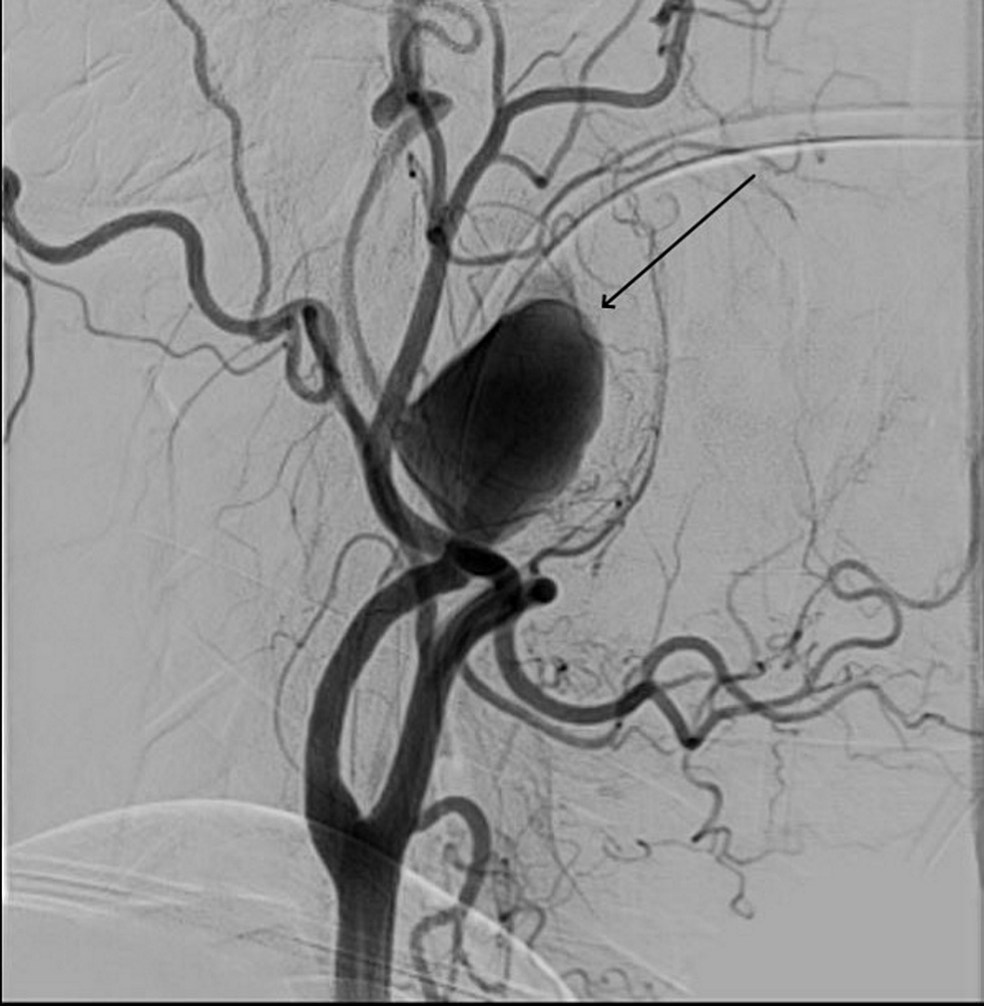
DSA showing large partially thrombosed wide-necked cervical ICA aneurysm DSA: digital subtraction angiography; ICA: internal carotid artery

Hematological and biochemical investigations were within the normal range. Following discussion with the on-call neurology team, a CT angiography of the neck and brain was performed, which revealed aneurysmal dilatation of distal cervical segment of left ICA measuring 3.2 x 4.4 x 5.0 cm with the lateral part of it opacified by contrast (1.3 x 2.1 x 3.0 cm) and the rest of the non-enhancing region suggestive of thrombus. The aneurysm was causing partial effacement of the airway. A digital subtraction angiography (DSA) showed a large partially thrombosed wide-necked cervical ICA aneurysm (Figure 1).

The aneurysm was large and caused mass effect resulting in multiple cranial nerve palsies, and hence urgent intervention was planned. The complete exposure of ICA was performed up to the skull base and the thrombus was removed. Vascular reconstruction was performed with a polytetrafluoroethylene graft (end-to-end anastomosis). The patient was discharged on postoperative day 12.

## Discussion

While assessing a patient with pulsatile tinnitus in the emergency department, thorough history and clinical examination can help to understand the likely pathology and narrow down the differential diagnosis. The medical history includes duration and severity of the tinnitus, history of trauma, associated auditory symptoms of hearing loss, vertigo, and neurologic symptoms of headache, visual disturbances, vomiting, and cranial nerve involvement. The clinical evaluation requires an otologic examination and a detailed neurological examination in addition to a local examination. Auscultation of the chest, neck, and skull should be performed to look for cardiac murmurs, cervical bruits, and objective tinnitus. Our patient had pulsatile tinnitus, lower cranial nerve deficit, a neck mass without bruit, and absence of ear mass on otologic examination. CT angiography revealed an aneurysm in the distal cervical segment of the ICA, which was partially non-enhancing due to the presence of the thrombus.

The most common site of extracranial ICA aneurysm is common carotid artery bifurcation and proximal ICA. The causes of ICA aneurysm include atherosclerosis, fibromuscular dysplasia, trauma (penetrating and blunt cervical trauma and hyperextension of the neck), iatrogenic lesions, infections, congenital defects, and irradiation arteritis [3,4]. In the pre-antibiotic era, most cervical ICA aneurysms were due to inflammatory causes like a tonsillar abscess. The trend later changed towards atherosclerosis, trauma, or postoperative carotid endarterectomy-induced aneurysms. Spontaneous congenital aneurysms have rarely been reported [5].

In our case, neck swelling was noticed about a month ago with the progressive increase in size. On further evaluation, we could not find any clue regarding traumatic or vasculitic etiology for this aneurysm. The patient may have been having a congenital swelling that was too small and had escaped his attention until recently.

The clinical presentations of extracranial ICA aneurysms vary from being asymptomatic and incidentally diagnosed to pulsatile mass in the neck, transient ischemic attacks, stroke, and cranial nerve involvement. Major complications involve compression of neurovascular structures, embolization, rupture, or ischemia [4]. The postulated etiology behind cranial nerve involvement is stretching of the nerve by the expanded artery or interruption of nutrient vessels supplying the nerve [6].

Aneurysms of the cerebral internal carotid or the vertebral arteries are a rare cause of pulsatile tinnitus. Pulsatile tinnitus as the sole manifestation of an ICA aneurysm in the paraclinoid portion has been reported [7]. Still, it is surprisingly rare for extracranial ICA aneurysms to clinically manifest as pulsatile tinnitus.

In this index case, the peritonsillar swelling was not evaluated as an extension of neck swelling but was mistaken for peritonsillar abscess and drained. Iatrogenic trauma led to pseudoaneurysm formation with thrombus, causing a rapid increase in size, and the resulting pressure effects caused disabling tinnitus with gradual paralysis of multiple cranial nerves. There was no bruit over the swelling; however, an absence of bruit does not rule out vascular etiology as it can be thrombosed. These procedures can be life-threatening for some patients in the absence of prompt evaluation and treatment.

## Conclusions

An aneurysmal swelling mistaken for peritonsillar abscess can lead to iatrogenic trauma resulting in serious complications like neurological deficits and thrombus formation. A prompt diagnosis requires detailed history and examination. Among the plethora of neurological manifestations, tinnitus may be the presenting manifestation of extracranial ICA aneurysm in rare cases. Thus, pulsatile tinnitus may be a warning sign for suspecting a potentially serious underlying disease, and a high index of suspicion is required to avoid iatrogenic disasters.
